# Assessing health-related quality of life in Chinese children and adolescents with cancer: validation of the DISABKIDS chronic generic module (DCGM-37)

**DOI:** 10.1186/s12885-021-07910-9

**Published:** 2021-02-27

**Authors:** Hasan Alelayan, Lizhu Liang, Rui Ye, Jiangnan Meng, Xiaoyan Liao

**Affiliations:** 1 Nursing Department of Zengcheng Branch, Nanfang Hospital, Southern Medical University, No. 28 Innovation Avenue, Zengcheng, Guangzhou, 511300 China; 2 School of Nursing, Southern Medical University, No. 1838 Guangzhou Avenue North, Guangzhou , 510515 China; 3 Pediatric Department, Nanfang Hospital, Southern Medical University, No. 1838 Guangzhou Avenue North, Guangzhou, 510515 China

**Keywords:** Children, Adolescents, Cancer, Health-related quality of life, Validation

## Abstract

**Background:**

With increasing cancer incidence and decreasing cancer mortality, there is a growing need for a valid and culturally adapted tool to measure health-related quality of life in children with cancer. This study validated the DISABKIDS Chronic Generic Module (DCGM-37) in Chinese children and adolescents with cancer.

**Methods:**

The DCGM-37 was translated and adapted for use in China following the guidelines from its copyright holders. In total, 140 children and adolescents with cancer and their guardians were included in this cross-sectional study. Internal consistency and test-retest reliability were evaluated. Convergent validity was examined using Pearson correlation between the DCGM-37 and the PedsQL 4.0 Generic Core Scale. Dimensionality was clarified using exploratory factor analysis. Discriminant validity was evaluated by comparing DCGM-37 scores by sex, age, family income, and clinical characteristics.

**Results:**

Internal consistency (Cronbach’s alpha 0.91) and test–retest reliability were good (intraclass correlation coefficient 0.87, 95% confidence interval 0.73–0.94). Strong correlations between the DCGM-37 and the PedsQL 4.0 (r = 0.83) suggest good convergent validity. Six factors explained 51.94% of the total variance. Children with leukemia scored higher than those with sarcoma in all subscales (effect size ranged from 0.39 to 0.83), especially the “social exclusion” subscales (effect size 0.83). Small to moderate differences (effect size ranged from 0.38 to 0.58) were observed by sex, age, and family income. Neither floor nor ceiling effects were observed.

**Conclusion:**

The DCGM-37 is reliable and valid for measuring health-related quality of life in Chinese children and adolescents with cancer.

**Supplementary Information:**

The online version contains supplementary material available at 10.1186/s12885-021-07910-9.

## Background

The latest childhood cancer report showed that global cancer incidence was 15.58 (per 100,000 population) in children and adolescents aged 0–19 years [1]. Since 1975, overall pediatric cancer incidence has increased slightly by 0.7% per year [2, 3]. In contrast, overall pediatric cancer mortality has reduced by 68% in children and 63% in adolescents over the past four decades [4]. In China, the cancer registration data show the same trend. For example, pediatric cancer morbidity was 8.71 (per 100,000 population) in 2015, down from 11.35 in 2009 [5]. Remarkable advances in cancer treatments have led to a transformation from an acute to a chronic care model for pediatric cancer, especially for hematologic malignancies [6]. Consequently, there is an increasing need to understand and promote health-related quality of life (HRQoL) for children and adolescents with malignant tumors [7].

Cancer therapies, such as chemotherapy, radiotherapy, and surgery, may affect many aspects of children’s lives, including physical, social, and emotional [8]. Previous studies suggest many physicians tend to underestimate symptom severity and overestimate overall health status of cancer patients [9]. Evaluation of HRQoL is crucial to appreciate how a new therapy has affected patients’ lives in both solid and hematological malignancies, especially for children [10]. The routine assessment of HRQoL may help monitor patients’ treatment burden, promptly identify patients at high risk of poor adherence behavior, improve symptom management [4], and develop specific supportive strategies to improve patients’ HRQoL. However, only a limited number of tools have been developed and validated to assess HRQoL from children and adolescents with cancer.

The Pediatric Quality of Life Inventory (PedsQL) [11] and the DISABKIDS [12] are two of the most commonly used instruments designed to evaluate HRQoL among children and adolescents with chronic conditions. Both these instruments contain a chronic generic module and disease-specific modules and share the same advantages; for example, having parallel versions for children and their proxies and having age-appropriate versions. The PedsQL was translated into Chinese in 2011 [13]. Unfortunately, that study observed a relatively large percentage of missing data for items reflecting “school functioning” using the PedsQL in Chinese samples, potentially resulting from cultural differences [13]. The DISABKIDS was cross-nationally developed by the European DISABKIDS project using a standardized translation guideline [14]. It is therefore a promising tool to compare HRQoL outcomes across countries and cultures.

The Chronic Generic Module in the DISABKIDS, abbreviated as DCGM-37, contains 37 items and addresses general aspects of children’s and adolescents’ lives [15]. As far as we know, only one previous study has tested its psychometric properties in children (age range 7–16 years old) undergoing treatment for cancer [7], and there is no Chinese version yet.

## Material and methods

### Aim

In this study, we aimed to examine the psychometric properties of the Chinese version of the DCGM-37 for assessing HRQoL among Chinese children and adolescents with cancer.

### Study design

This study was cross-sectional in design.

### Participants and setting

We recruited children and adolescents diagnosed with cancer and their proxies from a 2500-bed tertiary hospital in Guangzhou, China, using convenience sampling. The inclusion criteria for participation in the study were (1) children aged 8–18 years, (2) children diagnosed with cancer and undergoing active treatment, and (3) able to understand Mandarin. The exclusion criteria were (1) children with chronic diseases such as diabetes or asthma, (2) children with cognitive problems or psychiatric disorders identified from their medical records, (3) absence of a proxy, and (4) refusal or withdrawal of the agreement to participate.

The Medical Ethics Committee of Nanfang Hospital (NFEC-202006-K6–01) reviewed and approved this study. Before the survey, the fourth author explained the study’s purposes and procedures to all eligible children, adolescents, and parents. They were also told that the participants’ personal and medical information would be stored confidentially and reported anonymously. After obtaining written consent from the participants or their proxies, the participants and their proxies were asked to complete the DCGM-37 and PedsQL 4.0 Generic Core Scale. Two weeks after the first questionnaire, a convenience sample of 30 pairs of participants were asked to complete a retest questionnaire.

### Instruments

#### The DISABKIDS chronic generic module (DCGM-37)

The DCGM-37 contains 37 items. Its six subscales address six aspects of HRQoL from children’s and adolescents’ perspectives: independence (autonomy), physical limitation (functional limitations), emotions (emotional concerns), social exclusion (stigma and feeling left out), social inclusion (acceptance of others), and treatment (perceived impact of treatment) [12]. Participants scored each item on a five-point Likert-type scale, which rated the frequency of their behaviors or feelings during the past 4 weeks. Answers were 1 = never, 2 = seldom, 3 = quite often, 4 = very often, 5 = always. Within each subscale, the raw scores for each item were transformed to a 0–100 scale: 1 = 100, 2 = 75, 3 = 50, 4 = 25, 5 = 0; and then summed. Higher scores indicated better HRQoL. The DCGM-37 has demonstrated good internal consistency and construct validity in children with chronic conditions [15].

#### PedsQL 4.0 generic Core scale

The PedsQL 4.0 Generic Core Scale includes 23 items divided into four subscales: physical functioning, emotional functioning, social functioning, and school functioning. Each item is scored on a five-point response scale: 0 = never a problem; 1 = almost never a problem; 2 = sometimes a problem; 3 = often a problem; 4 = almost always a problem. The raw scores for each item are reverse-scored and transformed to a 0–100 scale, with higher scores indicating a better HRQoL. The PedsQL 4.0 Generic Core Scale has been tested in Chinese children with cancer [16]. We used the PedsQL 4.0 Generic Core Scale for children (8–12 years old) and adolescents (13–18 years old) to test the convergent validity of the DCGM-37 for children and adolescents (8–18 years old).

### Translation and pre-test

The DCGM-37 is a previously published questionnaire [7, 17]. The translation and linguistic validation for the DGCM-37 followed the DISABKIDS group’s guidelines [14]. The English version of the DGCM-37 was translated into Chinese by two bilingual medical experts whose mother tongue is Chinese. A panel of experts on pediatric nursing and methodology compared the two forward-translated versions to create the reconciled forward translation, and resolve ambiguities and discrepancies. An independent bilingual medical expert undertook back-translation, and we compared the original and back-translated versions. A pre-test was conducted among six children (8–12 years old, half boys and half girls), six adolescents (13–18 years old, half boys and half girls), and eight parents to evaluate comprehensibility and acceptance of the items in the Chinese version of the DCGM-37. A researcher carried out face-to-face interviews with the Children, adolescents, and their parents to establish whether (1) all the items were easy to understand, (2) any relevant issues were missing, and (3) the response categories were appropriate for each item. Answers were recorded. Finally, we sent the Chinese version back to the DISABKIDS group.

### Statistical analysis

Descriptive statistics were reported as frequencies and percentages for categorical variables and means and standard deviations (SDs) for continuous variables. We examined the internal consistency, Guttman’s split-half reliability, and test–retest reliability of the DCGM-37 using Cronbach’s alpha, split-half reliability coefficient, and the intraclass correlation coefficient (ICC). Test–retest reliability was estimated at after a 2-week interval to avoid memory bias. We also used ICC and paired t-tests to test the concordance and difference between DCGM-37 scores reported by children and their parents. An ICC between 0.61 and 0.80 indicates good or substantial agreements, and above 0.81 suggests excellent concordance [18]. The percentages of the minimum and maximum values were calculated to examine the ceiling and floor effects.

We explored the dimensionality of the DCGM-37 using exploratory factor analysis with Varimax rotation and principle component extraction. The Kaiser–Meyer–Olkin (KMO) test and Bartlett’s test of sphericity were used to verify sampling adequacy and satisfactory factor structure. Each retained factor had eigenvalues > 1. Items with a factor loading of 0.40 or greater were considered acceptable [18].

The correlation coefficients between the DCGM-37 and PedsQL 4.0 Generic Core Scale were calculated using Pearson correlation to examine the convergent and divergent validity of the DCGM-37. A correlation coefficient of greater than 0.5 was considered to be a strong correlation, 0.35–0.5 moderate, and less than 0.35 weak [18]. We required a moderate-to-strong correlation between the DCGM-37 and the PedsQL 4.0 Generic Core Scale for convergent validity. We hypothesized that a weak correlation coefficient would be present between the “emotion” subscale of the DCGM-37 and the “school functioning” of the PedsQL 4.0 Generic Core Scale. The PedsQL cancer module mainly focuses on symptoms (such as pain, nausea, and worry) specific to pediatric cancers. It does not encompass essential domains for measuring general health-related quality of life in children, such as physical functioning and social functioning. Therefore, we did not use the PedsQL cancer module to examine convergent validity of the DCGM-37.

We tested discriminant validity by comparing the DCGM-37 scores by sex, age, family income, and clinical characteristics. The participants were divided into two age groups: 8- to 12-year-olds and the 13- to 18-year-olds [12]. Effect size was expressed as Cohen’s d, and calculated for all comparisons to highlight the clinical importance of the differences. A Cohen’s d value of 0.20–0.50 was considered to be a small difference, 0.51–0.80 medium, and larger than 0.80 large [19]. We used SPSS version 25.0 (IBM Corp., Armonk, NY, USA) for all analyses. *P* < 0.05 was considered statistically significant.

We used AMOS version 24.0 (IBM Corp.) for separate confirmatory factor analysis for previously presumed domains (e.g., the mental, social, and physical domains) to evaluate their model adequacy [20]. A goodness-of-fit index (GFI) value of 0.90 or greater was considered to indicate an adequate model [20]. A comparative fit index (CFI) value of 0.90 or greater was considered to indicate a good global fit between a theoretical model and the data [21]. A value lower than 0.08 for the Root Mean Square Error of Approximation (RMSEA) was considered acceptable [21].

## Results

### Participants

In total, 140 children with cancer and their proxies completed the questionnaires. The average time to complete the instruments was approximately 15 min. The mean age of the proxies was 43.2 (±5.7) years. In total, 79.3% of proxies were mothers, and 17.1% were fathers. Table 1 shows the characteristics of the participants.

**Table 1:** Sociodemographic and clinical characteristics of the participants

Variables	Self-report (*n* = 140)	Proxy-report (*n* = 140)
Parents, n (%)	/	135 (96.4)
Children (8–12 years), n (%)	79 (56.4)	/
Adolescents (13–18 years), n (%)	61 (43.6)	/
Age, years, Mean ± SD	11.7 ± 2.5	43.2 ± 5.7
Girl, n (%)	66 (47.1)	111 (79.3)
Medical resource utilization, n (%)		/
Oncology outpatient	91 (65)	/
Pediatric oncology inpatient	49 (35)	/
Family monthly Income, n (%)		/
> 8001 Yuan	/	3 (2.1)
5001–8000 Yuan	/	39 (27.9)
3000–5000 Yuan	/	53 (37.9)
< 3000 Yuan	/	43 (30.7)
Unanswered	/	2 (1.4)
Diagnoses, n (%)		
Acute lymphoblastic leukemia	59 (42.1)	/
Acute myeloid leukemia	21 (15.0)	/
Non - Hodgkin’s Lymphoma	18 (12.9)	/
Hodgkin’s Lymphoma	8 (5.7)	/
Rhabdomyosarcoma	10 (7.1)	/
Osteosarcoma	17 (12.1)	/
CNS tumor	7 (5.0)	/

*CNS* central nervous system

### Concordance between self-report and proxy-report scores

ICCs between self-report and proxy-report scores for all subscales ranged from 0.65 to 0.75 (Table 2), indicating substantial concordance between the children and parents on children’s HRQoL. However, the mean reported scores in four of the six subscales (e.g., independence, emotion, social inclusion, and social exclusion) were significantly higher from the children than their parents (Table 2).

**Table 2:** Floor effect, ceiling effect, and reliability for the Chinese DISABKIDS Chronic generic module

Subscales	N	Mean	SD	ICC ^a^	Cronbach’s *a*	Split-half	%Floor	%Ceiling	N ^b^	ICC ^b^
**Mental domain**										
Independence	140/140	59.1/55.7*	16.6/18.3	0.75	0.80/0.85	0.74/0.82	0/0	0/0	30/30	0.72/0.79
Emotion	138/139	56.9/53.8*	14.5/14.5	0.65	0.78/0.78	0.75/0.78	0.7/0	0/0	30/30	0.80/0.82
**Social domain**										
Social inclusion	139/140	60.4/53.8*	14.7/17.7	0.72	0.75/0.81	0.70/0.75	0/0	0/0	30/30	0.73/0.87
Social exclusion	139/140	65.6/60.4*	13.9/14.7	0.68	0.75/0.82	0.79/0.81	0/0	0/0	30/30	0.81/0.85
**Physical domain**										
Physical limitation	140/140	55.0/52.4	17.6/18.6	0.70	0.80/0.84	0.76/0.79	0/0	0.7/0	30/30	0.77/0.81
Treatment	139/140	56.9/55.7	16.8/18.0	0.73	0.82/0.80	0.80/0.76	0/0	1.4/0	30/30	0.71/0.86
**Total score**	140/140	59.0/55.3*	11.3/12.9	0.85	0.91/0.93	0.77/0.84	0/0	0/0	30/30	0.87/0.89

Data were presented as self-report / proxy-report. *DISABKIDS* disabled children’s quality-of-life measure, *ICC* Infraclass correlation coefficients, *SD* standard deviation. ^a^ICC between the child- and proxy-report scores; ^b^for test-retest reliability. * *P* < 0.05

### Item analysis

The item-subscale correlation coefficients were greater than 0.30 for all the items (see supplementary file Table S1), suggesting acceptable conceptual equivalence of the instrument. Subscale intercorrelations indicate the degree to which the pairs of subscales measure the same thing. Pearson correlations showed moderate to strong subscale intercorrelations (self-report: 0.36–0.50; proxy-report: 0.39–0.61) and strong correlations between subscales and total scores (self-report: 0.69–0.76; proxy-report: 0.72–0.80) (see supplementary file Table S2).

### The reliability of the DGCM-37

The Cronbach’s alpha coefficients were excellent (self-report: 0.91; proxy-report: 0.93) for the total scores of the DGCM-37. The Cronbach’s alpha coefficients for the six subscales ranged from 0.75 to 0.82 for the self-report data and from 0.78 to 0.85 for the proxy-report data (Table 2). The Guttman split-half reliability coefficients were acceptable (self-report: 0.77; proxy-report: 0.84). ICCs across two-time points were good for all six subscales (Table 2) and total scores (children: 0.87; 95% confidence interval [CI] 0.73–0.94; parents: 0.89; 95%CI 0.77–0.95), indicating good test-retest reliability of the instrument. As shown in Table 2, no floor or ceiling effects were observed.

### The validity of the DGCM-37

#### Dimensionality

The exploratory factor analysis identified six factors for both self-report and proxy-report data (Table 3). The KMO test (self-report: 0.80; proxy-report: 0.85) and Bartlett’s test of sphericity (self-report: χ2 = 1938.38, *P* < 0.01; proxy-report: χ2 = 2509.12, P < 0.01) were satisfied. In line with the original design, items 1–6 loaded onto the “independence” factor; item 7–12 loaded onto the “physical limitation” factor; item 13–19 loaded onto the ‘emotion’ factor; item 20–25 loaded onto the “social exclusion” factor; item 26–31 loaded onto the “social inclusion’ factor, and item 32–37 loaded onto the “treatment” factor (see Table 3).

**Table 3:** the results of EFA for the DISABKIDS Chronic generic module

Item	Factor 1 (Treatment)	Factor 2 (Physical limitation)	Factor 3 (Emotion)	Factor 4 (Independence)	Factor 5 (Social exclusion)	Factor 6 (Social Inclusion)
A1.1				0.77/0.55		
A1.2				0.70/0.63		
A1.3				0.51/0.76		
A1.4				0.63/0.71		
A1.5				0.62/0.59		
A1.6				0.61/0.61		
A2.7		0.67/0.54				
A2.8		0.7/0.62				
A2.9		0.66/0.47				
A2.10		0.73/0.79				
A2.11		0.42/0.46				
A2.12		0.64/0.79				
A3.13			0.60/0.64			
A3.14			0.47/0.56			
A3.15			0.75/0.66			
A3.16			0.62/0.49			
A3.17			0.70/0.48			
A3.18			0.56/0.70			
A3.19			0.60/0.50			
A4.20					0.53/0.67	
A4.21					0.65/0.72	
A4.22					0.61/0.53	
A4.23					0.49/0.50	
A4.24					0.80/0.83	
A4.25					0.60/0.66	
A5.26						0.76/0.77
A5.27						0.64/0.8
A5.28						0.73/0.40
A5.29						0.51/0.56
A5.30						0.67/0.61
A5.31						0.44/0.05
A6.32	0.69/0.56					
A6.33	0.78/0.65					
A6.34	0.52/0.49					
A6.35	0.66/0.76					
A6.36	0.53/0.61					
A6.37	0.84/0.65					
Eigenvalue	3.43/3.23	3.32/3.64	3.31/3.10	3.25/3.35	3.01/3.68	2.91/3.33
% of total variance	9.27/46.56	18.23/19.79	27.17 /54.96	35.96/28.83	44.08 /9.95	51.94/37.82

Data were presented as child-report /proxy-report. *EFA* exploratory factor analysis

The summary of confirmatory factor analysis is shown in supplementary file Table S3. Using the data reported by the children, the physical (GFI = 0.92, CFI = 0.95, and RMSEA = 0.06) and mental (GFI = 0.92, CFI = 0.97, and RMSEA = 0.04) domains had high goodness of fit values. However, the social domain showed unsatisfactory model fit (GFI = 0.87, CFI = 0.85, and RMSEA = 0.09). We used modification indices in Amos version 25.0, and added error covariance to this model. The model fit improved (GFI = 0.91, CFI = 0.93, and RMSEA = 0.06). Using data reported by parents, the social domain (Model 0) had good goodness of fit values (GFI = 0.92, CFI = 0.94, and RMSEA = 0.06), but the physical (GFI = 0.88, CFI = 0.90, and RMSEA = 0.09) and mental (GFI = 0.89, CFI = 0.92, and RMSEA = 0.07) domains (Model 0) had unsatisfactory model fit. After error covariance correction, the model fit of both physical (GFI = 0.91, CFI = 0.94, and RMSEA = 0.07) and mental (GFI = 0.92, CFI = 0.97, and RMSEA = 0.04) domains improved.

#### Convergent and divergent validity

The total scores for the DCGM-37 were positively correlated with those for the PedQ 4.0 Generic Core Scale (self-report: r = 0.83, *P* < 0.001; proxy-report: r = 0.87, P < 0.001). This suggested that the the DCGM-37 had good convergent validity. There were moderate correlations between subscales of the DCGM − 37 and the PedQ 4.0 Generic Core Scale, including “physical limitation” vs. the “physical functioning”, “emotion” vs. “emotional functioning” and “social exclusion and inclusion” vs. “social functioning” (see Table 4).

**Table 4:** Correlations between the DCGM-37 and the PedsQL 4.0 Generic core scale

DCGM-37	PedsQL 4.0 Generic core scale					
	Physical Functioning	Emotional Functioning	Social Functioning	School Functioning	Psychosocial Health Summary Score	Total score
Independence	0.45*/0.48*	0.49*/0.50*	0.34*/0.36*	0.40*/0.42*	0.53*/0.57*	0.55*/0.59*
Physical limitation	0.74*/0.76*	0.47*/0.45*	0.45*/0.38*	0.57*/0.60*	0.64*/0.64*	0.64*/0.76*
Emotion	0.35*/0.52*	0.68*/0.67*	0.56*/0.40*	0.27*/0.47*	0.62 */0.69*	0.55 */0.69*
Social exclusion	0.41*/0.42*	0.47 */0.54*	0.49*/0.51*	0.46*/0.41*	0.64*/0.65*	0.64*/0.62*
Social inclusion	0.40*/0.59*	0.35*/0.53*	0.59*/0.68*	0.35*/0.39*	0.57*/0.70*	0.55*/0.72*
Treatment	0.40*/0.46*	0.43*/0.51*	0.44*/0.41*	0.39*/0.38*	0.55*/0.57*	0.54*/0.59*
Total score	0.64*/0.71*	0.67 */0.70*	0.66*/0.60*	0.57*/0.59*	0.81*/0.84*	0.83*/0.87*

**D**ata were presented as self-report / proxy-report. *DCGM-37* Disabled Children’s Quality-of-Life Measure (DISABKIDS) Chronic Generic Module, *PedsQL 4.0* the Pediatric Quality of Life Inventory 4.0. Psychosocial Health Summary *Score* summary score of the Emotional, Social, and School Functioning. *P < 0.01

The lowest correlation was found between the “emotion” subscale of the DCGM-37 and the “school functioning” subscale of the PedQ 4.0 Generic Core Scale (r = 0.27, *P* < 0.01) in data reported by the children, suggesting acceptable divergent validity.

#### Discriminant validity

Boys tended to score slightly higher than girls on the “emotion” subscale (effect size [ES] = 0.38). Older adolescents tended to score slightly higher than younger children on the “independence” (ES = 0.37), “physical limitation” (ES = 0.40), and “treatment” (ES = 0.40) subscales (Table 5). Children from families with low monthly income worse HRQoL in the mental domain, including the “independence” (ES = 0.50) and “emotion” (ES = 0.58) subscales, and for their total score (ES = 0.58) than children from families with a higher monthly income. Children with leukemia scored higher than those with sarcoma in all subscales and on the total score (ES ranged from 0.39–0.83; see Table 5), especially on the “social exclusion” subscales (ES = 0.83).

**Table 5:** Comparisons of subscale scores for the self-report version of the DCGM-37 across gender, age group, economic status, and clinical characteristics

Groups	Independence	Physical limitation	Emotion	Social exclusion	Social inclusion	Treatment	Total score
**Gender**							
Boys (*n* = 74)	61.3 ± 16.7	57.3 ± 18.2	59.4 ± 15.8*	65.8 ± 13.7	60.1 ± 16.1	56.3 ± 18.4	60.1 ± 12.8
Girls (*n* = 66)	56.7 ± 16.1	52.4 ± 16.6	54.2 ± 12.4	65.3 ± 14.4	60.7 ± 13.2	57.6 ± 15.0	57.8 ± 9.4
Effect size	0.28	0.29	0.38	0.04	0.04	0.08	0.21
**Age group**							
Children (*n* = 79)	56.6 ± 18.2*	52.0 ± 16.9*	56.8 ± 14.7	64.0 ± 13.9	58.3 ± 16.1	54.0 ± 18.6*	57.0 ± 12.0*
Adolescents (*n* = 61)	62.4 ± 13.6	58.7 ± 17.7	57.2 ± 14.3	67.5 ± 13.9	62.9 ± 12.4	60.5 ± 13.5	61.0 ± 9.8
Effect size	0.37	0.40	0.03	0.26	0.32	0.40	0.42
**Monthly income**							
High (*n* = 41)	64.0 ± 15.8 *	58.9 ± 16.7	61.6 ± 14.9 *	67.1 ± 13.3	63.7 ± 16.1	60.7 ± 16.9	62.7 ± 11.7 *
Low (*n* = 97)	57.5 ± 16.5	53.6 ± 17.8	54.9 ± 13.9	64.7 ± 14.2	59.0 ± 14.1	55.0 ± 16.6	57.5 ± 10.9
Effect size	0.50	0.38	0.58	0.21	0.40	0.41	0.58
**Clinical characteristics**							
Leukaemias ^a^ (*n* = 80)	63.1 ± 16.2**	59.0 ± 16.0**	59.8 ± 15.0**	69.8 ± 11.7**	65.0 ± 13.0**	60.4 ± 14.8**	62.9 ± 10.4**
Sarcoma ^b^ (*n* = 27)	52.5 ± 15.7	47.3 ± 20.1	48.8 ± 13.7	57.7 ± 16.4	50.8 ± 13.6	46.0 ± 15.9	55.6 ± 9.7
Effect size	0.60	0.57	0.39	0.83	0.68	0.44	0.82

Subscale scores are presented as mean ± standard deviation. *DCGM-37* DISABKIDS generic chronic module 37. ^a^ Acute lymphoblastic leukemia and acute myeloid leukemia. ^b^ Osteosarcoma and Rhabdomyosarcoma ^*^P < 0.05; ^**^P < 0.01

## Discussion

We followed the DISABKIDS group guidelines to translate and culturally adapt the DISABKIDS Chronic Generic Module (DCGM-37) into Chinese and then examined its psychometric properties in Chinese children and adolescents with cancer. We observed satisfactory homogeneity, time stability, dimensionality, construct validity, and discriminant validity of the Chinese version of the DCGM-37. Our findings suggest that the instrument is psychometrically acceptable for assessing HRQoL in Chinese children and adolescents with cancer. To our knowledge, this study is the first attempt to develop and examine the psychometric properties of a Chinese version of the DCGM-37 for measuring HRQoL of Chinese children with cancer.

Previous studies focusing on children with a chronic condition reported moderate ceiling effects on children’s rating of the “physical limitation,” “social exclusion,” and “treatment” subscales of the DCGM-37 [17, 20]. In line with previous findings focusing on children and adolescents with cancer [7], we found no ceiling or floor effects, indirectly supporting the instrument’s sensitivity in children with cancer. We also found that children’s DCGM-37 ratings were higher than their parents for the mental (“independence” and “emotion” subscales) and social (“social exclusion” and “social inclusion” subscales) domains. This may be because parents and children tend to disagree about mental health issues, such as anxiety and depression [22]. The chronic and fatal nature of cancer means that parents might perceive that their children’s illness has a more severe impact than is actually the case. Parents’ psychological distress is closely related to their children’s perceived vulnerability and might translate into perceptions of a greater impact on their children [23].

In our study, all 37 items of the DCGM-37 were assigned to its six subscales, indicating good dimensionality. For the self-report data, the “independence” and “emotion” subscales fitted into the mental health domain, and the “physical limitation” and “treatment” subscales fitted into the physical health domain. However, the “social inclusion” and “social exclusion” subscales fitted into the social health domain only after error covariance correction. Social inclusion describes accepting others and building up positive social relationships, and social exclusion relates to stigma and the feeling of being left out. The weakness of model fitness in the social health domain in this study and studies using versions of the questionnaire in other languages [20] might be a consequence of instabilities or inadequacies in the instrument’s conceptual foundation.

Sex stereotypes about tolerance of discomfort affect the rating of emotional well-being [7, 24]. In Chinese culture, men have long been encouraged to tolerate physical discomfort [25]. We also observed that boys reported higher emotional well-being than girls. The adolescents in our study reported higher scores for independence, physical limitation, and treatment than children aged 8–12 years. Lower–income was closely related to a worse quality of life in children with cancer [23]. We found that mental health assessed using the DCGM-37 was consistently low in children from families with relatively low monthly incomes. We also found that children and adolescents with leukemia had higher HRQoL in all aspects of the DCGM-37measurement than those with sarcoma. Children with leukemia only receive chemotherapy, but children with sarcomas receive complex therapy, such as combined chemotherapy, radiotherapy, and surgery [26]. The latter group may therefore experience more side effects or complications [7]. Our findings suggest that the Chinese version of the DCGC-37 has excellent discriminant validity for measuring HRQoL in children and adolescents with cancer.

### Strengths and limitations

This study’s main strength is establishing convergent and discriminant validity for a Chinese version of the DCGM-37. However, the study also had several limitations. First, it was conducted in a single tertiary medical center. Second, convenience sampling might have introduced bias about participants’ clinical conditions (e.g., mainly hematologic malignancies in this study). Third, the sample size was relatively small for subgroup comparisons. It was also impossible to test the DCGM-37’s responsiveness for detecting small but important changes in HRQoL among children with cancer, because of the cross-sectional design. Future studies should address all these challenges with a larger sample from more facilities.

### Implications for nursing practice and future research

Children with cancer need a reliable and valid tool to assess their health-related quality of life accurately before any intervention can be adequately evaluated and administered. It is particularly useful for health professionals, family members, and teachers to understand and support health-related quality of life for children and adolescents with cancer throughout treatment. Few suitable instruments with known psychometric properties are available for use in mainland China. This study suggests that DCGC-37 is a reliable and valid instrument for assessing health-related quality of life in Chinese children and adolescents with cancer. We hope its use will help advance research and clinical care for Chinese-speaking populations. Health professionals or clinical researchers can use this instrument to evaluate pharmaceutical or non-pharmaceutical interventions in pediatric cancer.

## Conclusion

This study examined the psychometric properties of the Chinese version of the DISABKIDS Chronic Generic Module, and provided reasonable evidence that the instrument is a reliable and valid way to assess health-related quality of life in the Chinese population with pediatric cancer.

## Supplementary Information

**Additional file 1.**

## Data Availability

The datasets used or analysed during the current study are available from the corresponding author on reasonable request.

## Abbreviations

CFI: Comparative fit index
DCGM-37: The Chronic Generic Module of the DISABKIDS
GFI: Goodness-of-fit index
HRQoL: Health-related quality of life
ICC: Intraclass correlation coefficient
KMO: The Kaiser–Meyer–Olkin test
PedsQL: The pediatric quality of life inventory
RMSEA: The root mean square error of approximation
