# Supplementary material for: Assessing health-related quality of life in Chinese children and adolescents with cancer: validation of the DISABKIDS chronic generic module (DCGM-37)
Source: BMC Cancer. 2021 Feb 27;21:204. doi: 10.1186/s12885-021-07910-9 (PMC7913225; doi:10.1186/s12885-021-07910-9)
Supplement: Supplementary file 1 — Additional file 1. [file 12885_2021_7910_MOESM1_ESM.docx]

**Assessing health-related quality of life in Chinese children and adolescents with cancer: Validation of the DISABKIDS chronic generic module (DCGM-37)**

**Authors**

Hasan Alelayan ^a, b^, MSN, RN, Lizhu Liang ^a, b^, BSN, RN, Rui Ye ^a, b^, BSN, RN, JiangNan Meng ^c^, BSN, RN, Xiaoyan Liao ^a*^, PhD, RN

^a^ Nanfang Hospital, Southern Medical University, Nursing Department of Zengcheng Branch. No. 28 Innovation Avenue, Zengcheng, Guangzhou 511300, China.

^b^ Southern Medical University, School of Nursing, No. 1838 Guangzhou Avenue North, Guangzhou, 510515, China.

^c^ Nanfang Hospital, Southern Medical University, Pediatric Department. No. 1838 Guangzhou Avenue North, Guangzhou 510515, China.

**Corresponding Author**

Xiaoyan Liao, PhD, RN, Nanfang Hospital, Southern Medical University, Nursing Department of Zengcheng Branch, No. 28 Innovation Avenue, Zengcheng, Guangzhou 511300, China. E-mail: liaoxy@smu.edu.cn. Fax: + 86-020-6164-1188. Phone: + 86-186-6503-9967.

**Table S1.** Item-subscale correlations.

| Item | Independence | Physical limitation | Mental emotion | Social exclusion | Social Inclusion | Treatment |
| --- | --- | --- | --- | --- | --- | --- |
| A1.1 | **0.70/ 0.81** | 0.33/0.60 | 0.42/0.53 | 0.33/0.45 | 0.27/0.36 | 0.27/0.52 |
| A1.2 | **0.77/0.73** | 0.46/0.40 | 0.43/0.34 | 0.49/0.32 | 0.41/0.30 | 0.45/0.46 |
| A1.3 | **0.67/0.67** | 0.28/0.39 | 0.18/0.35 | 0.36/0.40 | 0.21/0.28 | 0.14/0.36 |
| A1.4 | **0.66/0.58** | 0.26/0.32 | 0.34/0.37 | 0.42/0.30 | 0.3/0.26 | 0.32/0.28 |
| A1.5 | **0.64/0.77** | 0.33/0.51 | 0.21/0.55 | 0.22/0.52 | 0.29/0.35 | 0.17/0.46 |
| A1.6 | **0.79/0.74** | 0.33/0.36 | 0.36/0.45 | 0.28/0.42 | 0.34/0.23 | 0.31/0.48 |
| A2.7 | 0.36/ 0.44 | **0.75/0.68** | 0.27/0.47 | 0.31/0.34 | 0.33/0.42 | 0.32/0.36 |
| A2.8 | 0.37/0.39 | **0.77/0.78** | 0.39/0.35 | 0.35/0.30 | 0.31/0.52 | 0.29/0.26 |
| A2.9 | 0.30/0.45 | **0.68/0.70** | 0.23/0.44 | 0.31/0.34 | 0.3/0.48 | 0.28/0.35 |
| A2.10 | 0.31/0.57 | **0.71/0.86** | 0.28/0.47 | 0.3/0.42 | 0.18/0.48 | 0.23/0.41 |
| A2.11 | 0.41/0.43 | **0.65/0.70** | 0.26/0.37 | 0.36/0.41 | 0.32/0.47 | 0.24/0.37 |
| A2.12 | 0.25/0.44 | **0.68/0.71** | 0.29/0.36 | 0.2/0.26 | 0.25/0.34 | 0.23/0.43 |
| A3.13 | 0.17/0.48 | 0.06/0.31 | **0.59/0.64** | 0.14/0.38 | 0.11/0.27 | 0.16/0.40 |
| A3.14 | 0.35/0.29 | 0.36/0.23 | **0.61/0.58** | 0.29/0.36 | 0.27/0.19 | 0.21/0.30 |
| A3.15 | 0.29/0.18 | 0.18/0.28 | **0.72/0.58** | 0.29/0.17 | 0.23/0.27 | 0.20/0.15 |
| A3.16 | 0.39/0.48 | 0.41/0.38 | **0.7/0.67** | 0.27/0.43 | 0.31/0.40 | 0.32/0.31 |
| A3.17 | 0.44/0.38 | 0.31/0.47 | **0.75/0.62** | 0.42/0.27 | 0.3/0.32 | 0.33/0.19 |
| A3.18 | 0.22/0.53 | 0.23/0.36 | **0.65/0.77** | 0.26/0.53 | 0.21/0.34 | 0.36/0.37 |
| A3.19 | 0.27/0.35 | 0.37/0.35 | **0.63/0.53** | 0.24/0.34 | 0.23/0.38 | 0.17/0.36 |
| A4.20 | 0.33/0.48 | 0.30/0.37 | 0.23/0.50 | **0.62/0.78** | 0.3/0.39 | 0.28/0.37 |
| A4.21 | 0.34/0.37 | 0.19/0.29 | 0.35/0.35 | **0.75/0.74** | 0.38/0.30 | 0.26/0.33 |
| A4.22 | 0.33/0.42 | 0.33/0.29 | 0.26/0.44 | **0.66/0.70** | 0.34/0.40 | 0.35/0.38 |
| A4.23 | 0.29/0.24 | 0.26/0.20 | 0.22/0.22 | **0.64/0.53** | 0.34/0.24 | 0.29/0.26 |
| A4.24 | 0.32/0.48 | 0.32/0.43 | 0.25/0.48 | **0.72/0.86** | 0.24/0.41 | 0.25/0.37 |
| A4.25 | 0.41/0.44 | 0.34/0.31 | 0.34/0.37 | **0.7/0.72** | 0.37/0.27 | 0.26/0.31 |
| A5.26 | 0.31/0.24 | 0.21/0.41 | 0.3/0.35 | 0.31/0.27 | **0.72/0.74** | 0.25/0.27 |
| A5.27 | 0.31/0.25 | 0.24/0.36 | 0.32/0.19 | 0.32/0.24 | **0.65/0.69** | 0.21/0.22 |
| A5.28 | 0.38/0.42 | 0.33/0.39 | 0.21/0.44 | 0.43/0.41 | **0.77/0.65** | 0.24/0.42 |
| A5.29 | 0.22/0.28 | 0.21/0.35 | 0.24/0.35 | 0.38/0.48 | **0.65/0.71** | 0.38/0.32 |
| A5.30 | 0.39/0.32 | 0.37/0.56 | 0.24/0.36 | 0.37/0.28 | **0.74/0.74** | 0.29/0.38 |
| A5.31 | 0.18/0.30 | 0.27/0.55 | 0.14/0.39 | 0.15/0.44 | **0.55/0.76** | 0.15/0.41 |
| A6.32 | 0.20/0.40 | 0.18/0.38 | 0.14/0.43 | 0.12/0.28 | 0.19/0.40 | **0.66/0.72** |
| A6.33 | 0.33/0.42 | 0.28/0.38 | 0.3/0.35 | 0.34/0.41 | 0.33/0.47 | **0.82/0.71** |
| A6.34 | 0.37/0.39 | 0.31/0.36 | 0.32/0.28 | 0.36/0.30 | 0.33/0.27 | **0.71/0.64** |
| A6.35 | 0.26/0.51 | 0.34/0.40 | 0.37/0.34 | 0.4/0.35 | 0.36/0.29 | **0.73/0.79** |
| A6.36 | 0.28/0.36 | 0.27/0.21 | 0.21/0.21 | 0.27/0.29 | 0.22/0.22 | **0.67/0.58** |
| A6.37 | 0.32/0.44 | 0.25/0.30 | 0.32/0.38 | 0.33/0.38 | 0.21/0.39 | **0.80/0.72** |

Note: Pearon correlation coefficients were presented as child-report/proxy-report.

**Table S2.** Correlations between subscales and total scores for the Chinese DISABKIDS Chronic generic module.

| Subscales | Independence | Physical limitation | Emotion | Social exclusion | Social inclusion | Treatment |
| --- | --- | --- | --- | --- | --- | --- |
| Independence | / | 0.47**/0.57** | 0.46**/0.53** | 0.50*/0.48** | 0.44**/0.39** | 0.40**/0.56** |
| Physical limitation | 0.47**/0.57** | / | 0.41**/0.47** | 0.43**/0.42** | 0.40**/0.61** | 0.37**/0.49** |
| Emotion | 0.46**/0.53** | 0.41**/0.47** | / | 0.41**/0.52** | 0.36**/0.45** | 0.38**/0.43** |
| Social exclusion | 0.50**/0.48** | 0.43**/0.42** | 0.41**/0.52** | / | 0.48**/0.46** | 0.42**/0.48** |
| Social inclusion | 0.44**/0.39** | 0.40**/0.61** | 0.36**/0.45** | 0.48**/0.46** | / | 0.38**/0.48** |
| Treatment | 0.40**/0.56** | 0.37**/0.49** | 0.38**/0.43** | 0.42**/0.48** | 0.38**/0.48** | / |
| Total score | 0.76**/0.78** | 0.73**/0.80** | 0.69**/0.73** | 0.73**/0.72** | 0.70**/0.75** | 0.70**/0.76** |

**Note:** data were Pearson’s r and were presented as self-report / proxy-report. **P < 0.01.

**Table S3.** Model fit statistics of Confirmatory Factor Analysis for the DISABKIDS Chronic generic module.

| Domain | Self-report (n=140) | | | | | |  | Proxy-report (n=140) | | | | |
| --- | --- | --- | --- | --- | --- | --- | --- | --- | --- | --- | --- | --- |
|  | Model | Df | CMIN/df | GFI | CFI | RMSEA |  | Df | CMIN/df | GFI | CFI | RMSEA |
| Mental | Model 0 (original) | 64 | 1.20 | 0.92 | 0.97 | 0.04 |  | 64 | 1.76 | 0.89 | 0.92 | 0.07 |
|  | Model 1 |  |  |  |  |  |  | 60 | 1.29 | 0.92 | 0.97 | 0.04 |
| Social | Model 0 (original) | 53 | 2.25 | 0.87 | 0.85 | 0.09 |  | 53 | 1.58 | 0.92 | 0.94 | 0.06 |
|  | Model 1 | 52 | 1.55 | 0.91 | 0.93 | 0.06 |  |  |  |  |  |  |
| Physical | Model 0 (original) | 53 | 1.45 | 0.92 | 0.95 | 0.06 |  | 53 | 2.15 | 0.88 | 0.90 | 0.09 |
|  | Model 1 |  |  |  |  |  |  | 51 | 1.76 | 0.91 | 0.94 | 0.07 |

**Note:** Model 0: original model. Model 1: error covariance correction model. CFI: comparative fit index; GFI: goodness of fit index; RMSEA: The Root Mean Square Error of Approximation; CMIN: Chi-square Value; Df: degree of freedom.
